# The influence of body composition on renal function in patients with coronary artery disease and its prognostic significance: a retrospective cohort study

**DOI:** 10.1186/s12933-016-0420-7

**Published:** 2016-08-02

**Authors:** Yong Peng, Hua Wang, Fei Chen, Fang-yang Huang, Tian-li Xia, Yan-biao Liao, Hua Chai, Peng-ju Wang, Zhi-liang Zuo, Wei Liu, Chen Zhang, Yi-jian Li, Yi-yue Gui, Mao Chen, De-jia Huang

**Affiliations:** Department of Cardiology, West China Hospital, Sichuan University, 37 Guoxue Street, 610041 Chengdu, People’s Republic of China

**Keywords:** Coronary artery disease, Renal function, Body composition, Prognosis

## Abstract

**Objective:**

We try to analyse the effect of renal functions on death in CAD patients with different body compositions.

**Methods:**

A retrospective analysis was conducted in 2989 consecutive patients with CAD confirmed by coronary angiography were enrolled and were grouped into two categories: basically preserved renal function (PRF) (eGFR ≥60 ml/min) and obviously reduced renal function (RRF) (eGFR <60 ml/min). The influence of renal insufficiency on mortality of CAD was detected in every tertile of body composition, including body mass index (BMI), body fat (BF) and lean mass index (LMI). The end points were all-cause mortality.

**Results:**

The mean follow-up time was 29.1 ± 12.5 months and death events occurred in 271 cases. The percentage of patients with RRF was positively correlated with BF and inversely correlated with the LMI, but no relationship to BMI. The survival curves showed that the risk of death was significantly higher in the RRF patients in all subgroups stratified using BMI, BF, or LMI (log rank test, all p < 0.001). The COX multivariate regression analysis showed that the risk of death was significantly higher in the RRF patients with high BF (HR 1.95, CI 1.25–3.05) and low LMI (HR 1.82, CI 1.19–2.79). Meanwhile, risk of death was significantly higher in RRF patients with a high BMI (HR 2.08, CI 1.22–3.55) or low BMI (HR 1.98, CI 1.28–3.08) but this risk was not significant in patients with a medium BMI (HR 1.12, 0.65–1.94). The subgroup analysis of patients with acute coronary syndrome (ACS) showed similar results.

**Conclusions:**

For patients with CAD, renal insufficiency was positively correlated with BF, inversely correlated with LMI, and unrelated to BMI. The effect of renal insufficiency on the risk of death of CAD was related to body composition.

## Background

Obesity is becoming one of the most important global health problems. According to the World Health Organization (WHO) survey in 2008, approximately 35 % of adults over the age of 20 were overweight worldwide, accounting for 34 % of men and 35 % of women in this age group, and 10 % of men and 14 % of women were obese [1]. In China, the overweight population grew by nearly 50 % from 1992 to 2008 [2]. Studies have shown that obesity is associated with a range of chronic diseases, such as hypertension, high cholesterol, diabetes, coronary artery disease (CAD), and chronic renal insufficiency [3–5]. Because obesity is a risk factor for both CAD and chronic renal insufficiency, patients with CAD are prone to obesity and renal insufficiency. A range of studies suggested that renal dysfunction was an independent risk factor for death in patients with CAD [6, 7]. Researchers have conducted numerous studies on the relationship between obesity and the outcome of CAD but have reached different conclusions. Recently, researchers have proposed the theory of the “obesity paradox” [8, 9], and studies have shown that the obesity paradox applies to chronic kidney disease in addition to CAD [10, 11]. At present, few studies have been conducted to investigate the effect of renal insufficiency on the outcome of coronary artery disease in patients with different body compositions.

In this study, we evaluated body fat with body composition-related parameters, such as the body mass index (BMI), body fat (BF), and lean mass index (LMI), and analysed the effect of renal functions on death in CAD patients with different body compositions.

## Methods

### Study population

The data source for this investigation was the West China Hospital CAD database. This single center database prospectively includes all the CAD or high risk patients undergoing angiography in West China Hospital affiliated to Sichuan University. For this analysis, we enrolled consecutive patients with CAD from July 2008 to January 2012 of the database. Patients with CAD were eligible for inclusion if they were restricted to participants with angiographic evidence of ≥50 % stenosis in ≥1 coronary vessels. In addition to the above angiographic criteria, patients with acute coronary symdrome (ACS) were eligible for inclusion if they had the following criteria: (1) ischemic chest discomfort that increased or occurred at rest; and (2) elevated cardiac troponin I levels (≥0.03 μg/L) or elevated cardiac troponin T levels (≥42 ng/L); and/or (3) new or presumably new electrocardiographic deviation in at least two contiguous leads [either pathologic Q waves (≥0.04 s in duration), ST segment dynamic horizontal/down-sloping depression ≥0.05 mV, or persistent ST segment elevation ≥0.1 mV in ≥2 contiguous precordial leads or ≥2 adjacent limb leads or new left bundle branch block (LBBB)]. The exclusion criteria included malignancies, pregnancy, end stage renal disease (ESRD) with hemodialysis or renal transplant and severe liver or hematological diseases. These inclusion and exclusion criteria were met by 3365 continuously enrolled CAD patients. After excluding patients with loss of follow-up (n = 287) or incomplete follow-up data (n = 89), 2989 patients were included in the data analysis. The study protocol was approved by the local institutional review boards in accordance with the Declaration of Helsinki. All subjects provided written informed consent before enrolment.

### Baseline characteristics

Demographic data, medical history, cardiovascular risk factor, vital signs at admission, medication at discharge, and final diagnosis were obtained from the patients’ electronic medical records and reviewed by a trained study coordinator. Blood sample were collected before angiography, and plasma biomarkers including liver and kidney function (including the admission SCr levels), blood glucose, serum lipid, etc. were analyzed in the department of Laboratory Medicine, West China hospital, accredited by the College of American Pathologists. Hypertension was defined as those with systolic blood pressure (SBP) ≥140 mm Hg and/or diastolic blood pressure (DBP) ≥90 mm Hg and/or those receiving antihypertensive medications. Diabetes mellitus (DM) was diagnosed in patients who had previously undergone dietary treatment for diabetes, had received additional oral antidiabetic or insulin medication or had a current fasting blood glucose level of ≥7.0 mmol/L or random blood glucose level ≥11.1 mmol/L. Patients received care according to the usual practice; treatment was not affected by participation in this study.

### Body composition assessment

During hospitalization, body height and weight of the patients were measured by nurses using standard methods. BMI was calculated as weight (kg) divided by the square of height (m^2^). BF was estimated using the Clínica Universidad de Navarra—Body Adiposity Estimator (CUN-BAE) equation: BF = −44.988 + (0.503 × age) + (10.689 × sex) + (3.172 × BMI) − (0.026 × BMI^2^) + (0.181 × BMI × sex) − (0.02 × BMI × age) − (0.005 × BMI^2^ × sex) + (0.00021 × BMI^2^ × age), where sex is replaced by 0 for male and 1 for female individuals [12]. This formula has been validated in a large population [13]. The LMI was calculated as follows: (1 − %BF) × BMI kg/m^2^ [14]. As no reference value has been recommended for Chinese population, we divided the study patients into three groups according to the tertiles of sex-specific BMI, LMI, or BF.

### Renal function assessment

SCr was finished before the angiography within first 24 h after admission and assessed by a nonkinetic alkaline picrate (Jaffe) method. The Modification of Diet in Renal Disease (MDRD) equation was used to estimate glomerular filtration rate (eGFR) in milliliters per minute per 1.73 m^2^ [15]. Patients were divided into two eGFR groups: the basically preserved renal function (PRF) group [normal or mildly impaired renal function corresponding to strata used to define chronic kidney disease (CKD) stages [16], eGFR ≥60 ml/min] and the obviously reduced renal function (RRF) group (moderately or severely impaired renal function, eGFR <60 ml/min).

### Follow-up and end points

The follow-up period ended on January 2013. Follow-up information was collected through contact with patients’ physicians, patients or their family. All data were corroborated with the hospital records. The primary end points in this study were all-cause mortality and the secondary end points were cardiovascular death, as documented in the database. Death was considered cardiac when it was caused by acute MI, significant arrhythmias, or refractory heart failure. Sudden unexpected death occurring without another explanation was included as cardiovascular death.

### Statistical analyses

We conducted the post hoc analysis on a retrospective basis. Baseline demographics and clinical characteristics were compared among patients categorized by the admission eGFR levels in two groups. Continuous variables are expressed as the mean ± standard deviation (SD), and categorical variables are reported as counts and percentages. Analysis of t test and Chi squared tests were used to test for differences between groups for continuous and categorical variables, respectively. The comparisons of the distribution of eGFR <60 min/ml across the body composition tertiles were performed in Chi squared tests. To determine the association between renal function and all-cause mortality, Kaplan–Meier curves by eGFR levels were constructed in different body composition strata and examined using the log-rank test for comparison, respectively. Hazard ratios (HRs) and 95 % confidence intervals (CIs) were calculated based on Cox proportional hazards regression models, which was used to investigate the independent effect of renal function on the outcome events. Adjustments were made for the possible confounding effects of age, sex, medical history (pre-hypertension and pre-diabetes mellitus), heart function (Killip level), severity of CAD (left main artery and three vessel diseases), discharge medications [statin, angiotensin-converting enzyme (ACE) inhibitors or angiotensin-receptor blockers (ARBs) and beta-receptor blockers]. Two-sided *p* values of less than 0.05 indicated statistical significance. All analyses were performed with SPSS software (version 19.0).

## Results

A total of 2989 patients with coronary artery disease were included in this study. The patients were aged 64.5 ± 10.6 years on average, and 20.5 % were women. SCr was determined within 24 h after admission. The mean estimated glomerular filtration rate (eGFR) was 79.5 ± 22.9 ml/min, with an eGFR <60 ml/min in 506 patients (16.9 %). The baseline data are shown in Table 1. Significant differences were observed in the clinical characteristics between groups, with more severe renal insufficiency associated with an older age, female gender, and comorbidities such as hypertension, diabetes, cardiac dysfunction, and complex CAD.

**Table 1:** Baseline characteristics of the study population

Characteristics	eGFR			*p* value
	Total	<60 mL/min	≥60 mL/min	
No. of patients	n = 2989	n = 506	n = 2483	
Age, years	64.5 ± 10.6	70.4 ± 8.0	63.3 ± 10.7	<0.001
Gender, female, n (%)	614 (20.5)	162 (32.0)	452 (18.2)	<0.001
Body composition parameters				
BMI, kg/m^2^	24.2 ± 2.9	24.1 ± 2.8	24.2 ± 2.9	0.477
BF, %	28.1 ± 6.2	30.2 ± 6.6	27.7 ± 6.0	<0.001
LMI, kg/m^2^	17.3 ± 1.8	16.7 ± 1.9	17.4 ± 1.8	<0.001
Medical history				
Current smoking, n (%)	902 (30.2)	151 (29.8)	751 (30.2)	0.857
Pre-hypertension, n (%)	1628 (54.5)	352 (69.6)	1276 (51.4)	<0.001
Pre-diabetes mellitus, n (%)	653 (21.8)	154 (30.4)	499 (20.1)	<0.001
At admission				
Systolic blood pressure, mm Hg	130.3 ± 21.2	132.0 ± 24.1	129.9 ± 20.6	0.059
Diastolic blood pressure, mm Hg	76.3 ± 12.5	74.2 ± 13.4	76.7 ± 12.3	<0.001
Heart rate, beats/min	74.1 ± 13.9	76.3 ± 16.1	73.6 ± 13.4	0.001
Killip classification ≥ II, n (%)	344 (11.5)	93 (18.4)	251 (10.1)	<0.001
Laboratory values				
eGFR, ml/min/1.73 m^2^	79.5 ± 22.9	45.9 ± 12.4	86.3 ± 18.0	<0.001
Blood glucose, mmol/L	6.9 ± 3.1	7.7 ± 4.1	6.8 ± 2.8	<0.001
Total cholesterol, mmol/L	4.1 ± 1.1	4.0 ± 1.1	4.1 ± 1.1	0.222
Diagnosis				
ACS, n (%)	2163 (72.4)	382 (75.5)	1781 (71.7)	0.084
UA, n (%)	1533 (51.3)	257 (50.8)	1276 (51.4)	0.806
Severity of CAD				
Left main artery, n (%)	282 (9.4)	54 (10.7)	228 (9.2)	0.296
Three vessel diseases, n (%)	758 (25.4)	176 (34.8)	582 (23.4)	<0.001
Discharge medication				
Aspirin, n (%)	2760 (92.3)	432 (85.4)	2328 (93.8)	<0.001
Clopidogrel, n (%)	2670 (89.3)	428 (84.6)	2242 (90.3)	<0.001
Statin, n (%)	2678 (89.6)	432 (85.4)	2246 (90.5)	0.001
ACE inhibitors or ARBs, n (%)	1702 (56.9)	290 (57.3)	1412 (56.9)	0.854
Beta-receptor blockers, n (%)	1981 (66.3)	301 (59.5)	1680 (67.7)	<0.001

Data are expressed as mean ± SD or counts and percentages, as appropriate

*BMI* body mass index; *BF* body fat; *LMI* lean mass index; *eGFR* estimated glomerular filtration rate; *ACS* acute coronary syndrome; *UA* unstable angina; *CAD* coronary artery disease; *ACE* angiotensin-converting enzyme; *ARBs* angiotensin-receptor blockers

**Fig. 1:**
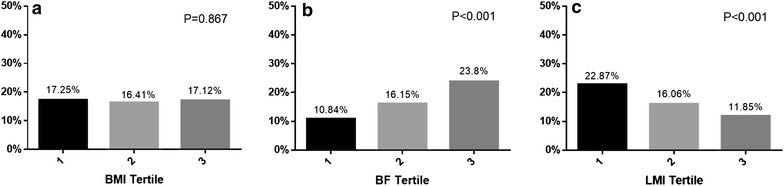
The proportion of patients with eGFR <60 min/ml across the BMI (**a**), BF (**b**) and LMI (**c**) tertiles. *eGFR* estimated glomerular filtration rate; *BMI* body mass index; *BF* body fat; *LMI* lean mass index

Figure 1 shows the relationship between renal insufficiency and body composition. For patients with CAD, the percentage of patients with RRF was positively correlated with BF and inversely correlated with the LMI; no significant relationship was observed between the BMI and the percentage of patients with renal insufficiency.

**Fig. 2:**
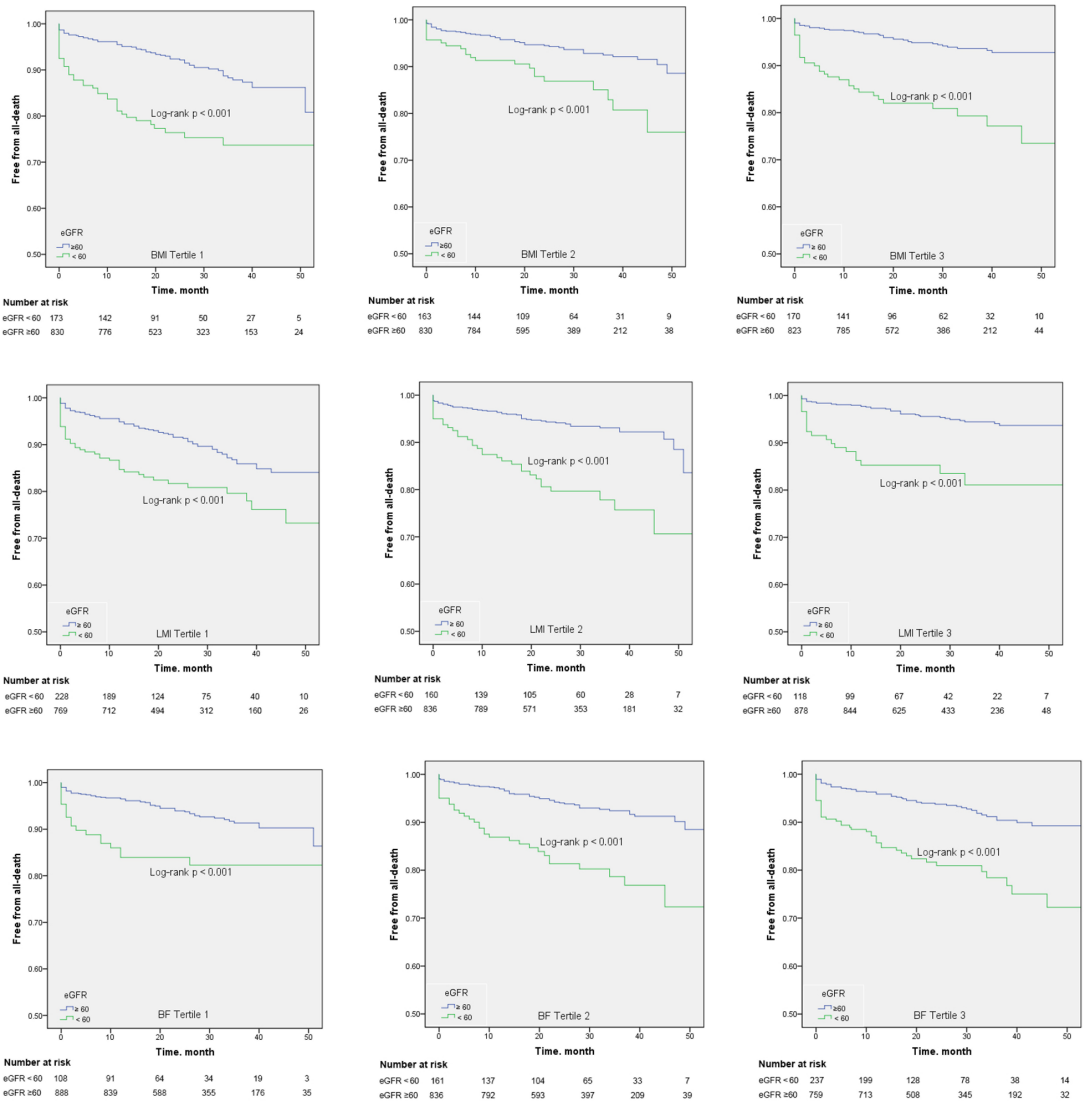
Kaplan-Meier plots according to the eGFR levels (min/ml) across the BMI, BF and LMI tertiles. *eGFR* estimated glomerular filtration rate; *BMI* body mass index; *BF* body fat; *LMI* lean mass index

All the patients were followed up for an average of 29.1 ± 12.5 months, during which 271 patients died (mortality rate: 9.1 %), including 150 who died of cardiovascular events (cardiac mortality rate: 5.0 %). The survival curves showed that the risk of death was significantly higher in the RRF patients in all subgroups stratified using BMI, BF, or LMI (log rank test, all p < 0.001; Fig. 2).

The COX multivariate regression analysis showed that the risk of death was significantly higher in the RRF patients with high BF (BF tertile 3) (HR 1.95, CI 1.25–3.05) and low LMI (LMI tertile 1) (HR 1.82, CI 1.19–2.79), whereas the risk of RRF for death was not significant in patients with low BF (BF tertile 1) (HR 1.64 0.92–2.93) and high LMI (LMI tertile 3) (HR 1.71, CI 0.91–3.20) after multivariable correction (Table 2). In contrast, RRF was a risk factor for death in patients with a high BMI (BMI tertile 3) (HR 2.08, CI 1.22–3.55) or low BMI (BMI tertile 1) (HR 1.98, CI 1.28–3.08) but this risk was not significant in patients with a medium BMI (BMI tertile 2) (HR 1.12, 0.65–1.94). The subgroup analysis of patients with ACS showed similar results (Table 2). Analysis of cardiac death (Table 3) showed that RRF was a more significant risk factor for death in patients with high BF (HR 1.90, CI 1.00–3.62) and low LMI (HR 2.46, CI 1.39–4.37), which was consistent with the findings of the analysis of all-cause death; however, the risk of RRF for cardiac death was only significant in patients with a low BMI but not those with a high or medium BMI. For ACS patients, the risk of RRF for cardiac death was only in patients with low LMI and was unrelated to cardiac death in all of the BMI and BF subgroups. This result might be related to the low number of cardiac death cases, which could have resulted in insufficient statistical power in the ACS subgroup.

**Table 2:** Hazard ratio for the incidence of all-cause mortality associated with renal function in the tertile subgroups of body composition

Outcomes	All-cause mortality			All-cause mortality			All-cause mortality		
	BMI			BF			LMI		
	Tertile 1	Tertile 2	Tertile 3	Tertile 1	Tertile 2	Tertile 3	Tertile 1	Tertile 2	Tertile 3
No. of patients	n = 1003	n = 993	n = 993	n = 996	n = 997	n = 996	n = 997	n = 996	n = 996
Total CAD patients (n = 2989), HR (95 % CI)									
No. of deaths	116	78	77	80	89	102	124	87	60
Unadjusted	2.91 (1.99–4.26)	2.30 (1.42–3.75)	4.03 (2.57–6.34)	2.75 (1.64–4.61)	3.15 (2.05–4.86)	2.97 (2.01–4.40)	2.10 (1.46–3.03)	3.32 (2.15–5.12)	3.93 (2.28–6.78)
Adjusted^a^	1.98 (1.28–3.08)	1.12 (0.65–1.94)	2.08 (1.22–3.55)	1.64(0.92–2.93)	1.57(0.95–2.60)	1.95(1.25–3.05)	1.82 (1.19–2.79)	1.69 (1.01–2.82)	1.71 (0.91–3.20)
ACS patients (n = 2163), HR (95 % CI)									
No. of deaths	89	63	63	57	69	89	100	67	48
Unadjusted	3.11 (2.03–4.80)	2.54 (1.50–4.29)	4.24 (2.58–6.98)	2.42 (1.30–4.49)	3.64 (2.24–5.90)	3.16 (2.08–4.79)	2.35 (1.57–3.50)	3.22 (1.96–5.29)	4.37 (2.42–7.90)
Adjusted^a^	1.67 (1.01–2.76)	1.02 (0.56–1.87)	1.97 (1.07–3.64)	1.11 (0.55–2.26)	1.39 (0.76–2.54)	1.93 (1.20–3.11)	1.74 (1.10–2.77)	1.14 (0.62–2.12)	1.84 (0.91–3.69)

Renal function was estimated by levels of the creatinine clearance and binary classified by ≥60 and <60 min/ml

*BMI* body mass index; *BF* body fat; *LMI* lean mass index; *HR* hazard ratio; *CI* confidence interval; *CAD* coronary artery disease; *ACS* acute coronary syndrome; *ACE* angiotensin-converting enzyme; *ARBs* angiotensin-receptor blockers

^a^Risk factors adjustment included age, sex, medical history (pre-hypertension and pre-diabetes mellitus), heart function (Killip level), severity of CAD (left main artery and three vessel diseases), discharge medication (statin, ACE inhibitors or ARBs and beta-receptor blockers)

**Table 3:** Hazard ratio for the incidence of cardiac mortality associated with renal function in the tertile subgroups of body composition

Outcomes	Cardiac mortality			Cardiac mortality			Cardiac mortality		
	BMI			BF			LMI		
	Tertile 1	Tertile 2	Tertile 3	Tertile 1	Tertile 2	Tertile 3	Tertile 1	Tertile 2	Tertile 3
No. of patients	n = 1003	n = 993	n = 993	n = 996	n = 997	n = 996	n = 996	n = 997	n = 996
Total CAD patients (n = 2989), HR (95 % CI)									
No. of cardiac deaths	65	45	40	50	47	53	68	50	32
Unadjusted	3.21 (1.95–5.30)	1.96 (1.01–3.79)	3.19 (1.68–6.06)	3.06 (1.63–5.77)	2.12 (1.12–4.01)	3.40 (1.98–5.83)	2.69 (1.66–4.34)	2.30 (1.25–4.21)	2.81 (1.26–6.27)
Adjusted^a^	2.26 (1.27–4.02)	0.85 (0.39–1.84)	0.90 (0.39–2.07)	1.66 (0.81–3.40)	0.72 (0.32–1.58)	1.90 (1.00–3.62)	2.46 (1.39–4.37)	0.89 (0.42–1.87)	0.77 (0.28–2.12)
ACS patients (n = 2163), HR (95 % CI)									
No. of cardiac deaths	50	38	35	36	38	49	56	42	25
Unadjusted	3.39 (1.92–5.97)	2.01 (1.00–4.05)	3.87 (1.98–7.56)	2.69 (1.26–5.73)	2.55 (1.29–5.05)	3.53 (2.01–6.18)	2.74 (1.62–4.65)	2.37 (1.23–4.57)	3.62 (1.56–8.41)
Adjusted^a^	1.85 (0.94–3.66)	0.69 (0.29–1.61)	1.00 (0.40–2.50)	1.23 (0.51–2.97)	0.56 (0.22–1.40)	1.80 (0.92–3.52)	1.99 (1.06–3.72)	0.63 (0.27–1.48)	0.99 (0.34–2.93)

Renal function was estimated by levels of the creatinine clearance and binary classified by ≥60 and < 60 min/ml

*BMI* body mass index; *BF* body fat; *LMI* lean mass index; *HR* hazard ratio; *CI* confidence interval; *CAD* coronary artery disease; *ACS* acute coronary syndrome; *ACE* angiotensin-converting enzyme; *ARBs* angiotensin-receptor blockers

^a^Risk factors adjustment included age, sex, medical history (pre-hypertension and pre-diabetes mellitus), heart function (Killip level), severity of CAD (left main artery and three vessel diseases), discharge medication (statin, ACE inhibitors or ARBs and beta-receptor blockers)

## Discussion

The study showed that renal insufficiency was positively correlated with BF, inversely correlated with LMI, and unrelated to BMI for patients with CAD. Moreover, the effect of renal insufficiency on the risk of death of CAD was related to body composition. The relationship between renal insufficiency and risk of mortality was more significant in CAD patients with higher BF and lower LMI, and with lower or higher BMI.

Some previous observational studies showed that obesity was a risk factor for chronic renal insufficiency [17–19], but other studies reached different conclusions [20–22]. Some researchers believed that the discrepancy arose because most of the previous studies used BMI as an indicator of obesity; however, BMI is not an accurate measure of obesity because it does not distinguish between body lean mass and body fat mass and body fat mass may play a major role in renal damage. Thus, recently researchers have proposed using BF and LMI to measure obesity [14, 23]. This study showed that renal insufficiency in patients with CAD was unrelated to BMI but was positively related to BF and inversely related to LMI, suggesting that BF and LMI were more effective measures than BMI when analysing the relationship between obesity and renal insufficiency. However, few studies have been conducted on this topic, and further exploration is warranted. The mechanism by which obesity causes renal damage is unknown. Possible mechanisms may be related to obesity-induced haemodynamic changes, a higher glomerular filtration burden, hormonal effects, and the activation of the renin-angiotensin-aldosterone-system (RAAS). Moreover, obese patients are prone to diabetes and hypertension, which indirectly cause renal damage [24, 25].

Renal insufficiency is an important risk factor for death and cardiovascular events in patients with CAD [6, 7, 26]. Furthermore, obese patients are susceptible to a range of cardiovascular risk factors, including hypertension, blood sugar disorders, dyslipidemia, and active inflammation [4]. Thus, cardiovascular risk factors may superimpose on one another in CAD patients complicated with obesity and renal insufficiency simultaneously. Obesity, especially the visceral adiposity would increase the risk of cardiovascular events and death in patients with renal insufficiency. The previous study found that visceral adiposity index is an optimal method to measure visceral adiposity to assess long-term CV outcomes and all-cause mortality in prevalent hemodialysis patients [27]. Epicardial adipose tissue accumulation in patients with CKD stage 3–5 increases the risk of CV events independent of general adiposity [28]. Visceral adiposity is associated with adverse cardiovascular outcomes for patients with CKD, while sarcopenia is common among patients with ESRD and is associated with higher mortality [29]. Considering the close relationship between obesity and renal insufficiency, accordingly, we have reason to speculate that renal function may perform different influence on the outcome of patients with different body compositions. Our study showed that renal insufficiency was a more significant risk factor for all-cause death and cardiac death in CAD patients with a higher body fat content (i.e., high BF and low LMI), suggesting that the fat mass may have a synergistic effect with renal insufficiency, thereby increasing the risk of death in CAD patients with renal insufficiency and obesity. How obesity causes cardiovascular disease is not entirely clear, but researchers believe that fat cells play an important role in this process as an endocrine organ [30]. Obese patients have a higher inflammation level and leptin resistance, which increase the risk of cardiovascular events [31, 32]. Moreover, obesity induces haemodynamic changes, increases the cardiac burden, induces cardiac remodelling, and affects cardiac functions [33].

Previous study found a U-shaped association of body mass index with inflammation and atherosclerosis in hemodialysis patients [34]. Interestingly, our study showed that renal insufficiency increased the risk of death of CAD in patients with a low BMI or high BMI but not in patients with a medium BMI (after multivariate correction), indirectly demonstrating that the BMI reflects the patient’s physical and nutritional status but not necessarily the body fat content. Patients with a high BMI are more likely to be obese, and thus, renal insufficiency may increase the risk of death in these patients, whereas patients with a low BMI are likely to have both a low fat body mass and low lean body mass (poor nutritional status); thus, renal insufficiency may more likely increase the risk of death in these patients. A previous cross-sectional study investigated the BMI and BF% (measured with air displacement plethysmography) in coronary artery disease (CAD) patients and showed that BMI failed to distinguish the fat mass from lean mass [35]. Our results indirectly support this opinion. For studies of the outcome of CAD, BF% and LMI may be more effective indicators than BMI for the risk of death.

This study has certain limitations. First, this study was a single-centre observational study. Despite multivariate correction, it was difficult to avoid all the confounding effect of (residual) selection bias absolutely. Moreover, in this single-centre study, all of the enrolled subjects were Chinese; thus, the potential for racial variation is unknown. Second, because the sample size of ACS subgroup was small relatively, the statistical power might have been inadequate in the analysis of cardiac death; thus, caution is needed when considering these results. In addition, the sample size of hemodialysis is small and we could hardly perform the analysis for this special subgroup. Considering the heterogeneity of patients with hemodialysis compared with patients without hemodialysis, we excluded the patients with hemodialysis in this study. Third, serum creatinine was collected only once after admission, and thus, measurement error could not be rule out definitely. Meanwhile, the serum creatinine levels might substantially change in some patients during hospitalization and the follow-up treatment after discharge. However, it is not always possible to repeat the serum creatinine test in an observational study, which is an inherent limitation of real world studies. Finally, this study was conducted in patients with CAD, and thus, it is unknown whether the results can be applied to renal insufficient patients without CAD.

## Conclusions

For patients with CAD, renal insufficiency was positively correlated with BF, inversely correlated with LMI, and unrelated to BMI. The effect of renal insufficiency on the risk of death of CAD was related to body composition. Renal insufficiency was a more significant risk factor in CAD patients with higher BF and lower LMI, and with lower or higher BMI.

## Abbreviations

ACE: angiotensin-converting enzyme
ACS: acute coronary syndrome
ARBs: angiotensin-receptor blockers
BF: body fat
BMI: body mass index
CAD: coronary artery disease
CI: confidence interval
eGFR: estimated glomerular filtration rate
ESRD: end stage renal disease
HR: hazard ratio
LMI: lean mass index
PRF: preserved renal function
RRF: reduced renal function
SCr: serum creatinine
SD: standard deviation
UA: unstable angina
